# Transcriptome profiling of anti-müllerian hormone treated preantral/small antral mouse ovary follicles

**DOI:** 10.18632/oncotarget.25572

**Published:** 2018-07-13

**Authors:** Zia ur Rehman, Faheem Ahmad Khan, Farmanullah, Hira Sajjad Talpur, Qing Liu, Shenhe Liu, Liguo Yang

**Affiliations:** ^1^ Key Laboratory of Agricultural Animal Genetics, Breeding and Reproduction, Education Ministry of China, College of Animal Science and Technology, Huazhong Agricultural University, Wuhan, People's Republic of China; ^2^ Department of Animal Health, Faculty of Animal Husbandry and Veterinary Sciences, The University of Agriculture, Peshawar, Pakistan; ^3^ The Center for Biomedical Research, Key Laboratory of Organ Transplantation, Ministry of Education, Ministry of Health, Tongji Hospital, Tongji Medical College, Huazhong University of Science and Technology, Wuhan, People's Republic of China; ^4^ College of Animal Science and Technology, Yangzhou University, Yangzhou, People's Republic of China

**Keywords:** AMH, DEGs, follicles, ovaries

## Abstract

The predisposition for the initiation of folliculogenesis in mammals including humans is programmed to start at fetal life and continues until reproductive capacity. The follicles grow from a pool of primordial follicles which retain the major functions in the entire reproductive life of a female. Anti-müllerian hormone (AMH), a glycoprotein belonging to the transforming growth factor-beta family, has an inhibitory effect on ovarian follicle development. The key regulatory target genes in primordial follicle development are of paramount importance in reproductive biology of female. A systems biology method was used to find regulatory genes performing critical role in primordial follicle development. A complete in-depth bioinformatics analysis was performed to investigate the changes in transcriptome of preantral to small antral mouse follicles treated for 12 h and 24 h with two different concentrations; 50 and 200 ng/ml of AMH, and thereby identify candidate genes in time and concentration manner. Firstly, we found differentially expressed genes that were time and concentration dependent in response to AMH. The network analysis of these differentially expressed genes provided new candidate genes and pathways associated with inhibitory action of AMH on the primordial follicle development. To further emphasize the function of AMH, the key identified genes’ protein-protein docking was analyzed and found the intracellular and extracellular protein-protein interaction. This study elucidates one of the novel mechanisms of AMH involvement in inhibition of ovarian follicle development which may lead to prolong productive life in female.

## INTRODUCTION

The mechanism of activation of primordial follicle to a healthy preantral follicle is of pronounced interest and their interpretation is critical requirement to use the primordial follicle and enhance its efficiency in mammals. The initiation of the follicular growth still needs investigation that how a follicle has a potential for continuing growth. It has been suggested previously that a follicle growth may occur due to a sophisticated balance between the stimulatory and inhibitory growth factors in ovary. Thus the activation of the follicle depends upon several factors in the microenvironment of each follicle [1]. The transition of primordial follicle to primary follicle involves the change in the histology of granulosa cells from round to cuboidal epithelial and increase in the oocytes diameter. With growth of follicle the granulosa and theca cell layers also increases, until ultimately a fluid filled antrum forms [2, 3]. With gradual development of the primary follicle it leaves the arrested pool of reproductive lifespan. The follicle depletion carries the female into cessation of reproductive life [4, 5]. The proportion of primordial follicle is fixed in pre reproductive life and the ovarian follicle pool is the major element for determining the reproductive life in mammals [6, 7]. The transition of primordial pool to primary follicle is regulated by certain paracrine and/or autocrine growth factors. The extracellular hormones/proteins have been investigated and found of critical importance in primordial follicular pool in female reproduction, Anti-müllerian hormone (AMH) performs inhibitory role in follicle transition [8] and chemokine which binds to functionally signaling G-protein-coupled receptors and complete their action [9]. It is also reported that oocytes of primordial and primary follicles express stromal derived factor -1 (SDF-1) (chemokine) and when ovaries were cultured with stromal derived factor-1 reveal decrease in follicular diameter as compared with control, suggesting an inhibitory effect of primordial to primary follicle transition [10].

Anti-müllerian hormone (AMH) is a member of transforming growth factor-β super family of growth factors and binds to its receptor-II (AMHRII). Initially it was known for the role of regression of Müllerian ducts in early fetal development in male embryo [11]. It is expressed in postnatal granulosa cells and helps in selection of developing follicles; it inhibits the development of primordial follicle into primary follicle pool and reduces the response of FSH to growing follicles [12]. The inhibitory role of primordial follicle transition needs further clarification and here we will focus on this study.

Anti-Müllerian hormone (AMH) plays an imperative role in folliculogenesis. It is one of the factors which regulate the kinetics of follicular development and inhibit the follicular transition for primordial to mature follicles [13]. Its exposure can decrease the expression of stimulatory factors and increase the expression of inhibitory factors and regulate the cellular signaling pathway resulting in the slowing down of primordial follicle development [14]. In AMH deficient mice large number of follicle development and high rate of oocyte degradation and atresia of follicle was observed due to low level of FSH which sustain the development of pre-ovulatory follicles demonstrating that AMH is critical for small-growing follicles [15]. It is a putative regulator of follicular atresia showing a time restrained expression which gives a platform for progression of normal folliculogenesis. It may affect some folliculogenesis affecting growth factors and enzymes. We speculate that AMH could be a crucial factor that can alter the normal mode of follicular atresia.

*In vitro* addition of recombinant AMH can preserve the primordial pool and shows inhibitory effect on the early follicular development by depressing the growth of follicular development in human as well as in mouse ovaries [16, 17]. It demonstrates that AMH acts as a negative paracrine response on the initiation of adjacent primordial pool and acts as a gatekeeper in controlling the initiation and depletion of primordial follicle pool [18]. A detailed in-silico bioinformatics analysis of the factors and cellular pathways altered by AMH can provide a clear understanding about the molecular control of primordial follicle development and is carried out in the present study to provide further evidence of AMH's role in primordial follicle development. The genes and pathways identified can be of profound interest in answering long standing questions regarding primordial follicle development that lays the foundation of female reproductive life throughout. Furthermore, the use of AMH identifies its importance as a therapeutic agent as and when it is required. The present study can be used as an engine in any further studies focusing AMH and its role in primordial follicle development.

## RESULTS

### Data processing and DEGs screening

**Figure 1:**
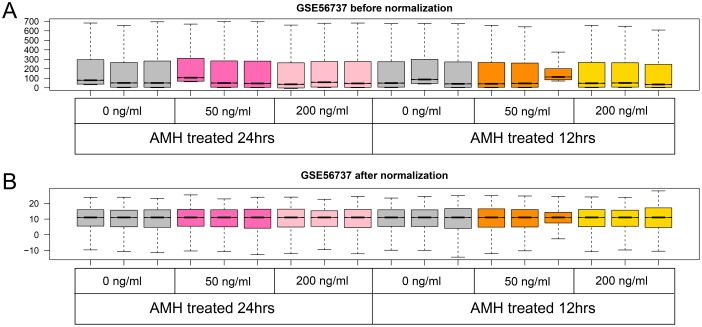
Boxplot of GSE56737 data preprocessing before (**A**) and after normalization (**B**). Grey boxes mean control samples of different time point and concentrations, hotpink and lightpink boxes mean 50 and 100 ng/ml AMH treated for 24 hours, orange and yellow boxes mean 50 and 100 ng/ml AMH treated for 12 hours.

**Figure 2:**
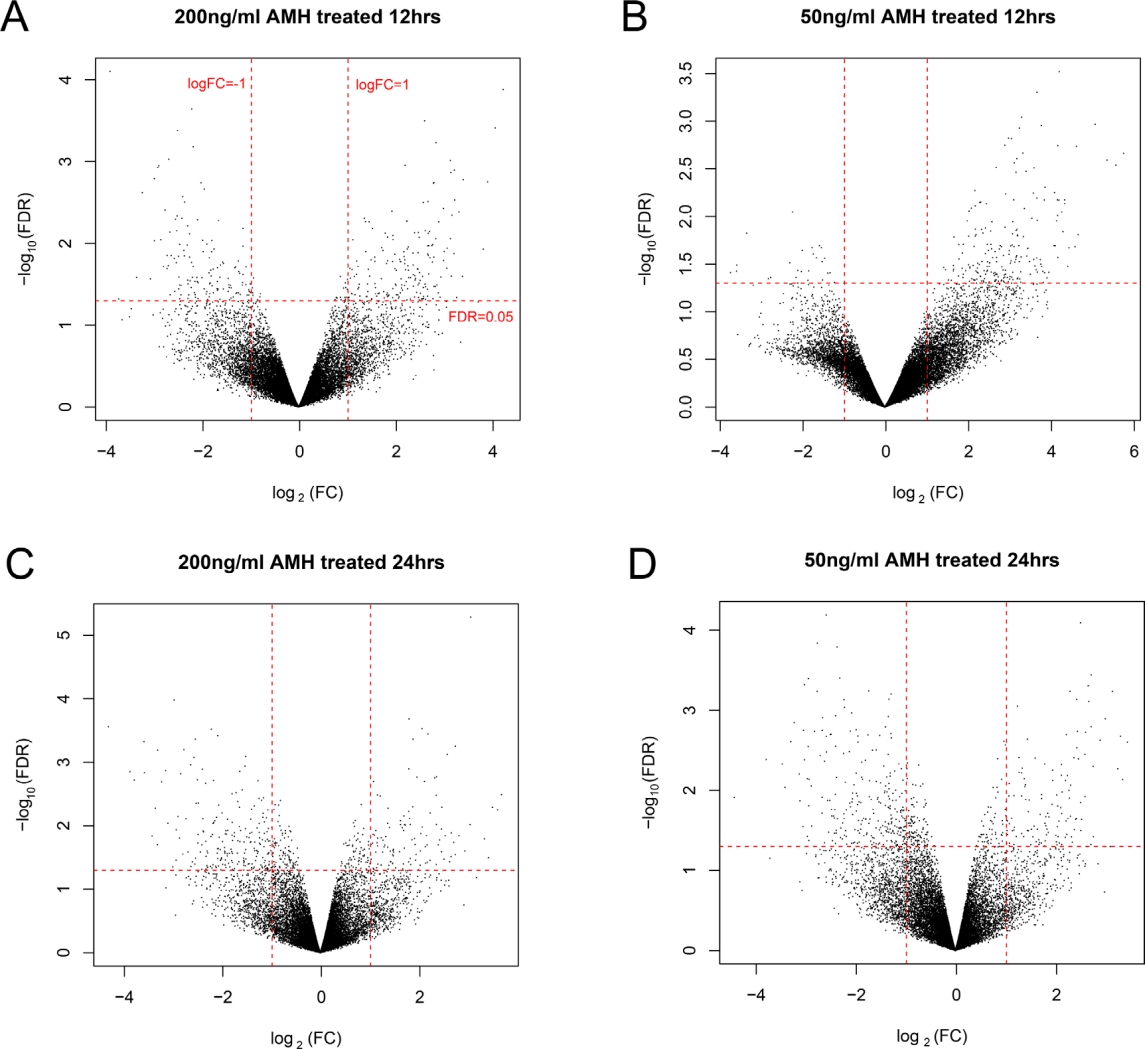
Volcano plot of DEGs in group a (**A**), b (**B**), c (**C**) and d (**D**), regions. Red horizontal dot line means FDR = 0.05 cutoff line, two red vertical dot lines mean logFC = 1 and logFC = -1 cutoff line.

We firstly normalized the dataset, see the preprocessing before and after box figures in Figure 1, and normalized expression data can be found in Supplementary Table 1. Based on the cut-off criteria, we screened 598, 571, 536 and 607 DEGs in group a, b, c and d, respectively. Volcano plots that illustrate the inclusion criteria for each region were shown in Figure 2. DEGs under a set of defined conditions lead us to the proposition of genes working in network to carry out a certain function, hence we figured out DEGs to further illustrate its role primordial follicle development. (DEGs list can be found in Supplementary Table 2).

### Hierarchical clustering and comparison analysis of selected DEGs in different groups

**Figure 3:**
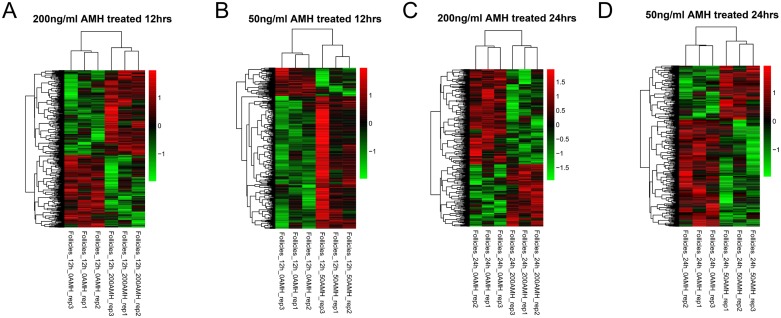
Hierarchical clustering of DEGs group a (**A**), b (**B**), c (**C**) and d (**D**).

**Figure 4:**
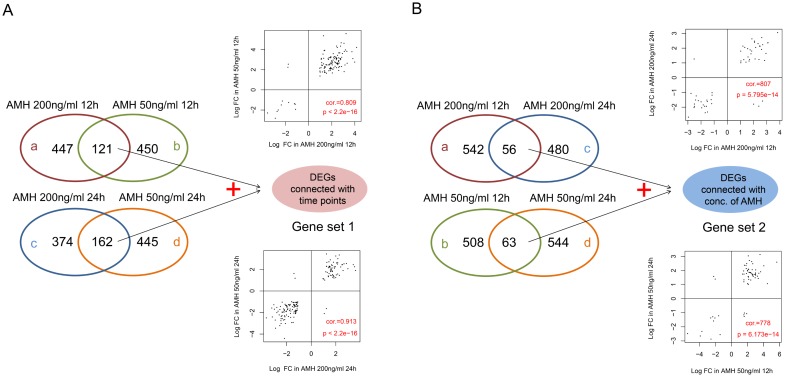
(**A**) Venn diagram of DEGs in a vs b and c vs d. Scatter-plot on the top means correlation between logFC of a and b; Scatter-plot on the bottom means correlation between logFC of c and d. (**B**) Venn diagram of DEGs in a vs c and b vs d. Scatter-plot on the top means correlation between logFC of a and c; Scatter-plot on the bottom means correlation between logFC of b and d. In the scatter-plot, cor refers to Pearson correlation coefficient, p refers to significance *p* value.

The DEGs networking explains how genes work together to bring out a certain phenotype under a set of defined conditions. It also leads towards the cellular signaling pathways that are important to carry out these functions. For this purpose we extracted DEG expressions from four different groups and drew hierarchical clustering heatmaps, as shown in Figure 3. It was clear that the samples in each group were divided into two types (AMH treated and control), which indicating that DEGs in each group had obvious different expression patterns. Then, we compare DEGs in four different groups, result was shown in Figure 4. When compared group a and b, they shared 121 DEGs, they had the same differential expression patterns in a and b, with a significant correlation coefficient of 0.809 (*p <* 2.2e-16); group c and d shared 162 DEGs, which had the same differential expression patterns in c and d with a significant correlation coefficient of 0.913 (*p <* 2.2e-16). We defined the 283 (121+162) DEGs as genes set 1, which were connected with time points. At the same time, when compared group a and c, they shared 56 DEGs, they had the same differential expression patterns in a and c, with a significant correlation coefficient of 0.807 (*p* = 5.795e-14); group c and d shared 63 DEGs, which had the same differential expression patterns in c and d with a significant correlation coefficient of 0.778 (*p <* 6.173e-14). We defined the 118 (56 + 63) DEGs as genes set 2, which were connected with concentration of AMH. DEGs related to time points (Gene set 1) and concentration of AMH (Gene set 2) were further analyzed. (Four parts of comparison overlapped between a and b, c and d, a and c, b and d, as well as DEGs in geneset 1 and 2, were listed in Supplementary Table 3).

### GO and KEGG pathway enrichment analysis for the Co-regulated DEGs

**Figure 5:**
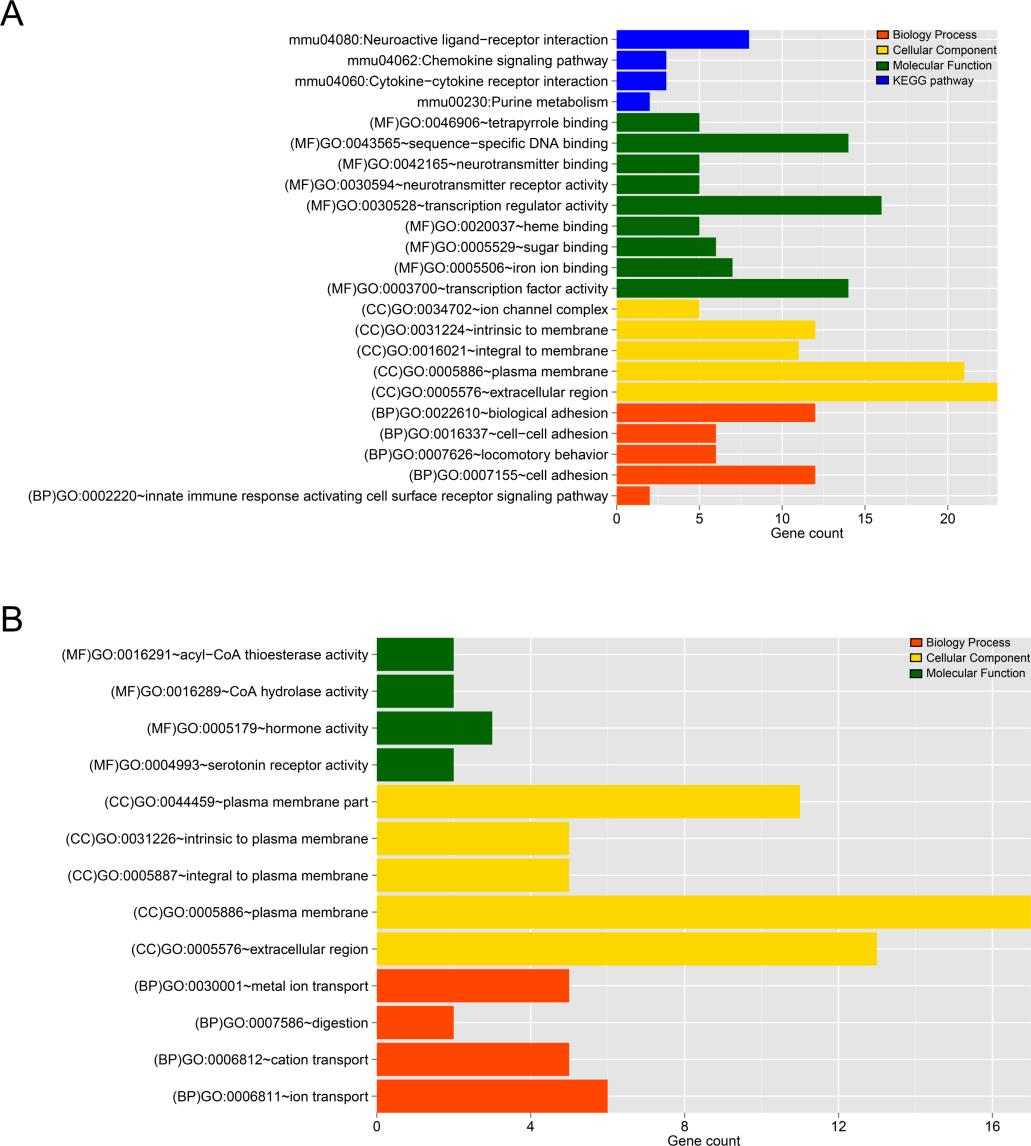
**(A)** The histogram of the category of enriched GO terms and KEGG pathways for the DEGs in gene set 1. (**B**) The histogram of the category of enriched GO terms and KEGG pathways for the DEGs in gene set 2. The horizontal axis represents the number of genes, red, yellow, green and blue mean Biology Process, Cellular Component, Molecular Function and pathways, respectively.

The DEGs identified were further enriched to a total of 19 (5 BP, 5 CC, 9 MF) and 13 (4 BP, 5 CC, 4 MF) significant related GO annotations were found for gene sets 1 and 2, respectively, listed in Tables 1 and 2. In gene set 1, enriched GO BPs were related to cell adhesion, biological adhesion, innate immune response and etc. Some genes, such as AZGP1, PODXL2, DSG1A, PCDHB18, CDH18, etc. were participated in term cell adhesion, which had the most enrichment significant *p* value and with the largest number of genes (23 genes were involved). At the same time, 4 pathways were enriched, there were 8 genes (GRM3, GABRR1, CHRM4, LTB4R1, DRD2, NMUR2, GALR2, HTR6) participating in the same pathway called Neuroactive ligand-receptor interaction. And CCR7 and CXCR2 were involved in two pathways: Chemokine signaling pathway and Cytokine-cytokine receptor interaction. The pathways altered due to AMH treatment at time point is shown in Figure 5A. While in gene set 2, KCNK15, SLC34A1, KCTD16, SLC30A8, 1300017J02RIK showed the same trend of taking part in three BP terms at the same time: metal ion transport, ion transport and cation transport (No significantly enriched pathways were found for DEGs in gene set 2). Using ggplot2 package in R, significant related GO annotations in gene set 1 and 2 were displayed in Figure 5.

**Table 1:** Enriched GOs and KEGG pathways for DEGs in gene set 1

Term	Count	*P* Value
(BP)GO:0007155~cell adhesion	12	0.0061064
(BP)GO:0022610~biological adhesion	12	0.0061862
(BP)GO:0002220~innate immune response activating cell surface receptor signaling pathway	2	0.0322812
(BP)GO:0016337~cell-cell adhesion	6	0.0441598
(BP)GO:0007626~locomotory behavior	6	0.0461861
(CC)GO:0005886~plasma membrane	21	6.75E-04
(CC)GO:0016021~integral to membrane	11	0.0191657
(CC)GO:0005576~extracellular region	23	0.0259966
(CC)GO:0031224~intrinsic to membrane	12	0.0272382
(CC)GO:0034702~ion channel complex	5	0.0465984
(MF)GO:0043565~sequence-specific DNA binding	14	3.22E-04
(MF)GO:0042165~neurotransmitter binding	5	0.0044747
(MF)GO:0030594~neurotransmitter receptor activity	5	0.0044747
(MF)GO:0003700~transcription factor activity	14	0.0063171
(MF)GO:0005529~sugar binding	6	0.0127776
(MF)GO:0020037~heme binding	5	0.0248372
(MF)GO:0046906~tetrapyrrole binding	5	0.0289048
(MF)GO:0030528~transcription regulator activity	16	0.0416291
(MF)GO:0005506~iron ion binding	7	0.0484739
mmu04080:Neuroactive ligand-receptor interaction	8	6.79E-04
mmu04062:Chemokine signaling pathway	3	0.0293552
mmu04060:Cytokine-cytokine receptor interaction	3	0.0427581
mmu00230:Purine metabolism	2	0.0461171

BP: Biology Process; CC: Cellular Component; MF: Molecular Function.

**Table 2:** Enriched GOs and KEGG pathways for DEGs in gene set 2

Term	Count	*P* Value
(BP)GO:0030001~metal ion transport	5	0.043398
(BP)GO:0006811~ion transport	6	0.046156
(BP)GO:0006812~cation transport	5	0.046858
(BP)GO:0007586~digestion	2	0.047563
(CC)GO:0005576~extracellular region	13	0.00493
(CC)GO:0005886~plasma membrane	17	0.013229
(CC)GO:0044459~plasma membrane part	11	0.029641
(CC)GO:0005887~integral to plasma membrane	5	0.04884
(CC)GO:0031226~intrinsic to plasma membrane	5	0.049853
(MF)GO:0004993~serotonin receptor activity	2	0.042361
(MF)GO:0005179~hormone activity	3	0.047403
(MF)GO:0016291~acyl-CoA thioesterase activity	2	0.045998
(MF)GO:0016289~CoA hydrolase activity	2	0.047441

BP: Biology Process; CC: Cellular Component; MF: Molecular Function.

### Co-expression network analysis for DEGs in gene set 1 and 2

**Figure 6:**
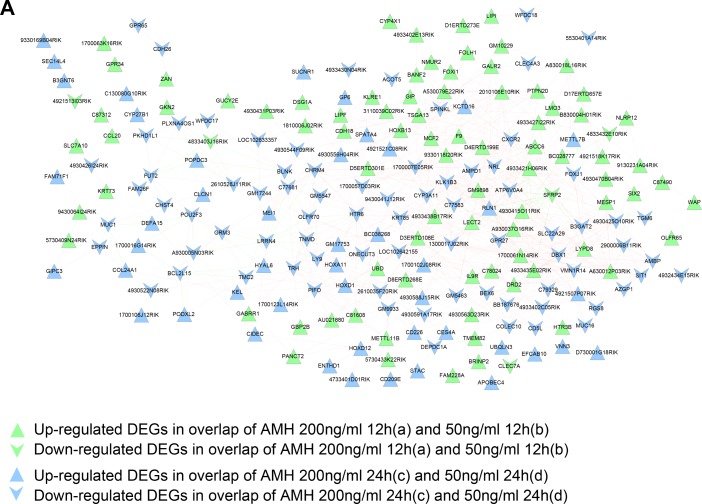

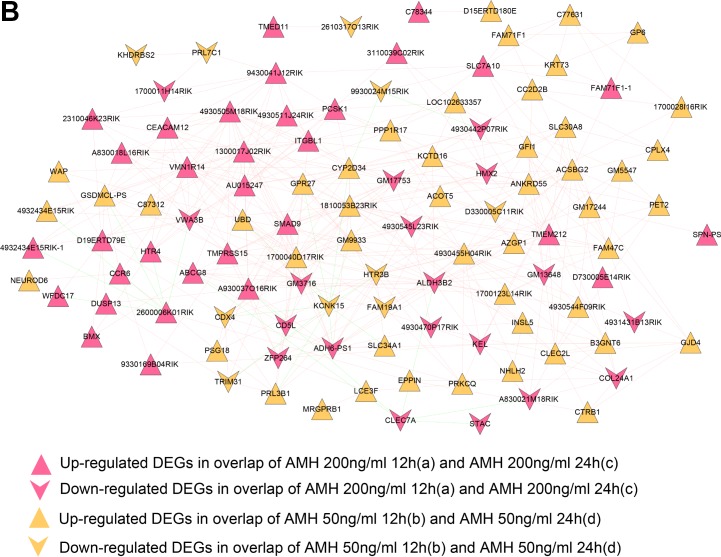
**(A)** Co-expression network of gene set 1. Vee and triangle nodes mean down and up regulated genes, green nodes mean gene nodes from overlap DEGs of a and b, blue ones mean gene nodes from overlap DEGs of c and d. (**B**) Co-expression network of gene set 2. Vee and triangle nodes mean down and up regulated genes, red nodes mean gene nodes from overlap DEGs of a and c, orange ones mean gene nodes from overlap DEGs of b and d. Green edges mean negative PCC connection, red edges mean positive PCC connection.

In a biological system several genes co-express along with each other and influencing the related functions, hence is important to screen out such genes for further functional studies, hence we developed a co-expression network for DEGs in gene set 1 and gene set 2. We calculated gene expression PCCs between every two genes, and kept gene pairs whose PCC score were over 0.8 to construct co-expression network of DEGs in gene set 1 and 2, respectively. We finally got 720 and 460 pairs of genes which meet 0.8 cutoff value as co-expression gene pairs in gene set 1 and 2 (gene pairs and their co-expression score were listed in Supplementary Table 4). Networks were shown in Figure 6. A total of 222 nodes (1down and 87 up regulated gene nodes from a and b overlap; 78 down and 56 up regulated gene nodes from c and d overlap) and 720 edges (59 negative and 661 positive connection) were included in Figure 6A network. While in co-expression network of gene set 2 shown in Figure 6B, there were 109 nodes (19 down and 30 up regulated gene nodes from a and c overlap; 10 down and 50 up regulated gene nodes from b and d overlap) and 460 edges (24 negative and 436 positive connection) involved.

### miRNA- DEGs-TF regulatory network construction

**Figure 7:**
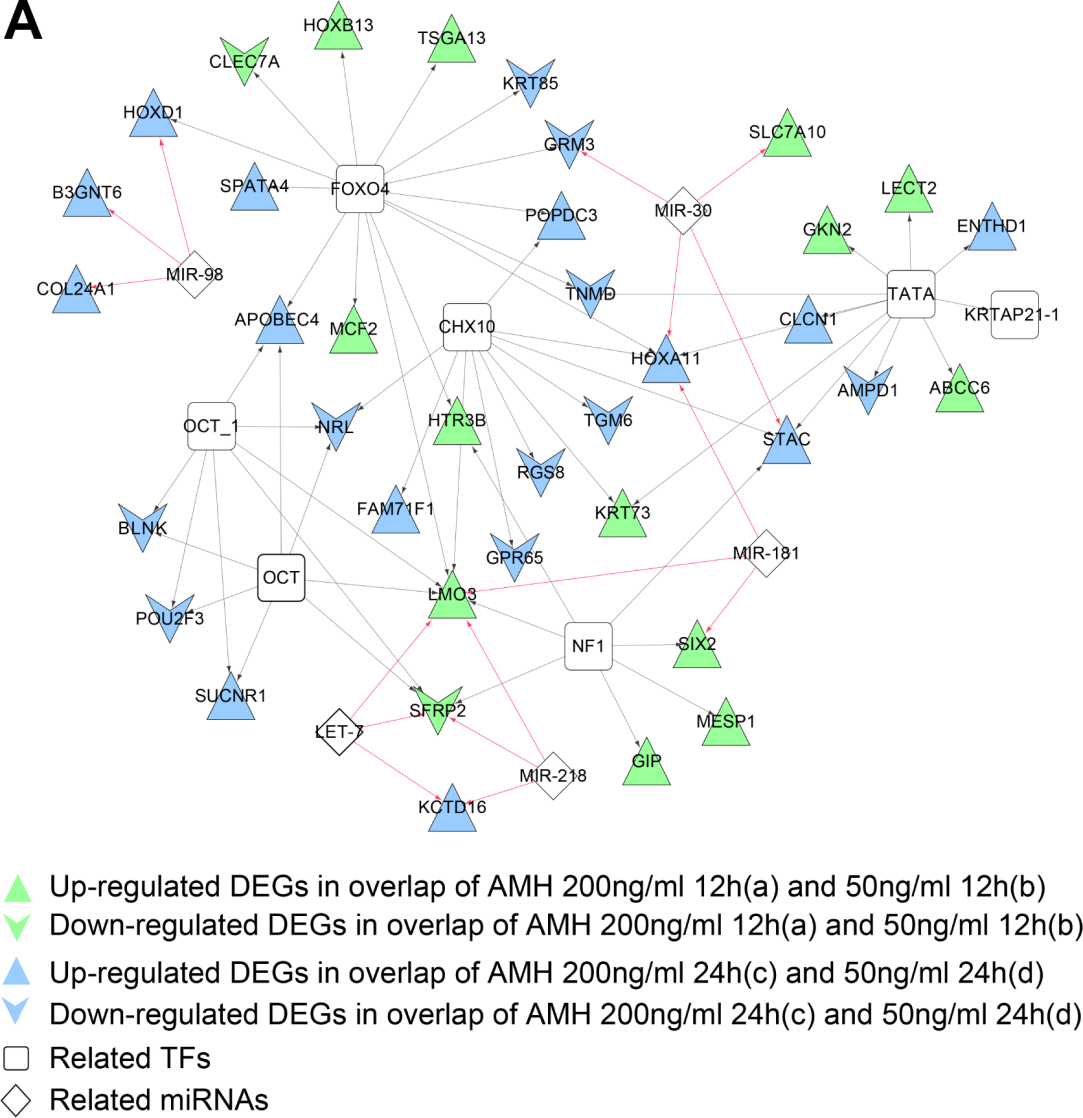

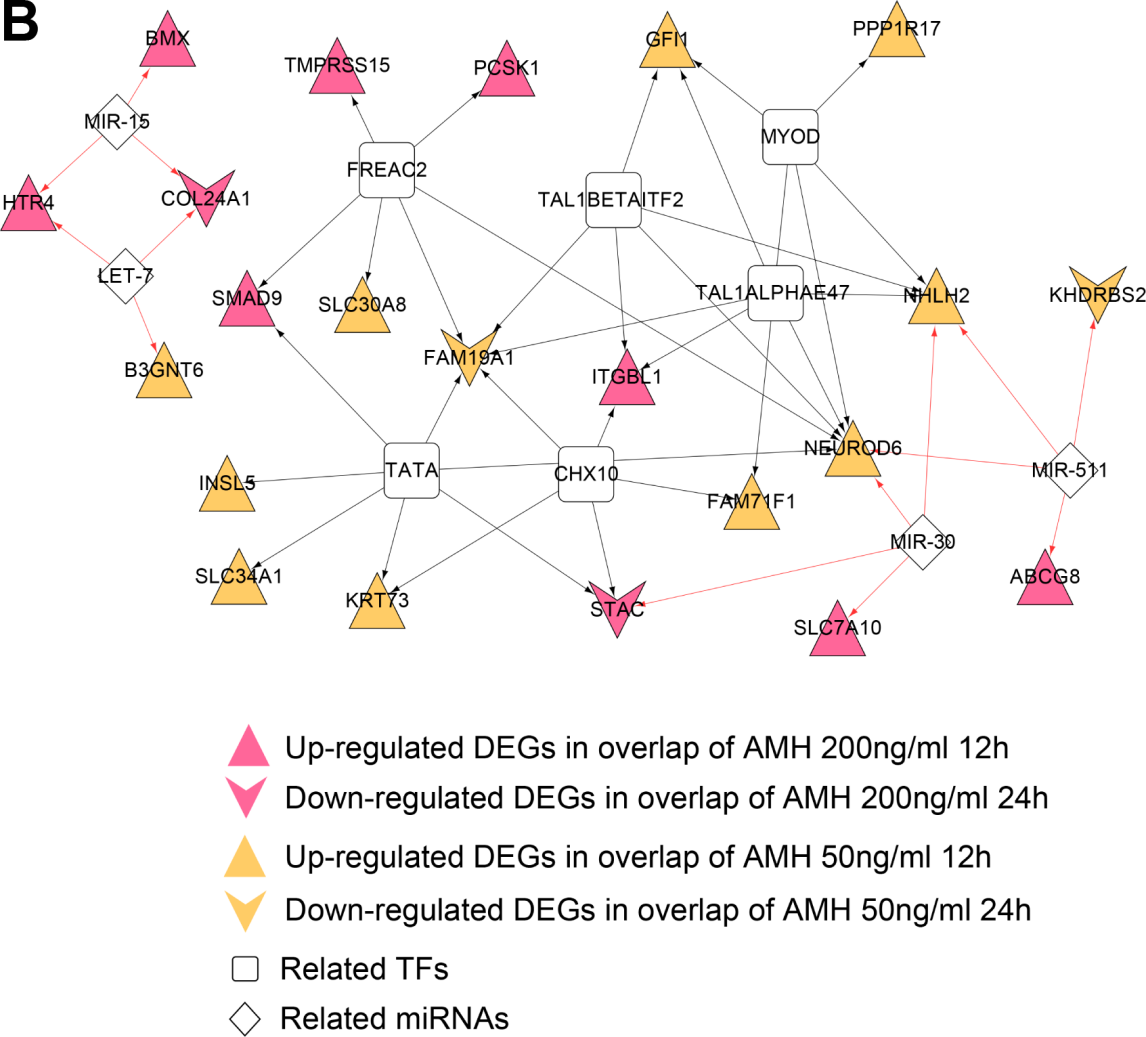
**(A)** miRNA-DEG-TF regulatory network of DEGs in gene set 1 co-expression network. Vee and triangle nodes mean down and up regulated genes, green nodes mean gene nodes from overlap DEGs of a and b, blue ones mean gene nodes from overlap DEGs of c and d. (**B**) miRNA-DEG-TF regulatory network of DEGs in gene set 1 co-expression network. Vee and triangle nodes mean down and up regulated genes, red nodes mean gene nodes from overlap DEGs of a and c, orange ones mean gene nodes from overlap DEGs of b and d. Black arrow lines mean TF-DEGs regulatory relationships, red arrow lines mean miRNA-DEGs regulatory relationships.

Micro RNAs affect the expression of genes and play critical role in its regulation. We screened totally, 16 and 14 miRNA and target regulatory relationships (involved 5 and 4 miRNAs), which were significantly related to DEGs in co-expression networks, were found in gene set 1 and 2 co-expression networks, related miRNAs of each network were listed in Table 3-1 and 3-2. At the same time, 56 and 33 TFs and target regulatory relationships (involved 6 and 6 TFs), which were significantly related to DEGs in co-expression networks, were found in gene set 1 and 2 co-expression networks, related TFs of each network were listed in Table 4-1 and 4-2. We then integrated miRNA and TF regulatory relationships and construct miRNA-DEGs- TFs regulatory network, which were shown in Figure 7. Figure 7A was consisted of 38 gene nodes (2 down and 13 up regulated gene nodes from a and b overlap; 10 down and 13 up regulated gene nodes from c and d overlap), 5 miRNAs and 6 TFs; while Figure 7B had 22 gene nodes (2 down and 8 up regulated gene nodes from a and c overlap; 2 down and 10 up regulated gene nodes from b and d overlap), 4miRNAs and 6 TFs. The Supplementary Table 5 shows the detail list of gene set 1 and 2 microRNA- TF network.

**Table 3-1:** Related miRNAs of DEGs in Figure 6A

microRNA	*P*-value	FDR	Gene
mmu_TGTTTAC,MIR-30	0.001378	0.02149	Grm3,Slc7a10,Stac,Hoxa11
mmu_AAGCACA,MIR-218	0.001612	0.03149	Sfrp2,Kctd16,Lmo3
mmu_CTACCTC,LET-7	0.001431	0.02149	Sfrp2,Kctd16,Lmo3
mmu_CTACCTC,MIR-98	0.001131	0.0149	Hoxd1,B3gnt6,Col24a1
mmu_TGAATGT,MIR-181	0.00223	0.0423	Six2,Lmo3,Hoxa11
			

**Table 3-2:** Related miRNAs of DEGs in Figure 6B

microRNA	*P*-value	FDR	Gene
mmu_AAAGACA,MIR-511	0.0004	0.0016	Abcg8,Nhlh2,Khdrbs2,Neurod6
mmu_TGTTTAC,MIR-30	0.0177	0.0354	Nhlh2,Slc7a10,Neurod6,Stac
mmu_CTACCTC,LET-7	0.028	0.0373	B3gnt6,Col24a1,Htr4
mmu_TGCTGCT,MIR-15	0.0841	0.0841	Bmx,Col24a1,Htr4

**Table 4-1:** Related TFs of DEGs in Figure 6A

TF	*P*-value	FDR	Gene
OCT_1	3.90E-05	0.0002	Nrl,Sfrp2,Apobec4,Sucnr1,Lmo3,Pou2f3,Blnk
OCT	3.00E-05	0.0002	Nrl,Sfrp2,Apobec4,Sucnr1,Lmo3,Pou2f3,Blnk
CHX10	0.0004	0.0014	Rgs8,Tgm6,Krt73,Fam71f1,Popdc3,Hoxa11,Nrl,Gpr65,Stac,Lmo3
TATA	0.0041	0.0118	Ampd1,Clcn1,Krt73,Abcc6,Gkn2,Tnmd,Hoxa11,Krtap21-1,Stac,Lect2,Enthd1
FOXO4	0.0099	0.0223	Grm3,Htr3b,Hoxb13,Hoxd1,Krt85,Popdc3,Tsga13,Tnmd,Mcf2,Hoxa11,Clec7a,Apobec4,Spata4,Lmo3
NF1	0.0118	0.0236	Six2,Htr3b,Sfrp2,Stac,Lmo3,Mesp1,Gip

**Table 4-2:** Related TFs of DEGs in Figure 6B

TF	*P*-value	FDR	Gene
TAL1ALPHAE47_01	6.67E-05	0.0005	Gfi1,Nhlh2,Neurod6,Itgbl1,Fam19a1
TAL1BETAITF2_01	6.81E-05	0.0005	Gfi1,Nhlh2,Neurod6,Itgbl1,Fam19a1
TATA_01	0.0058	0.0203	Krt73,Neurod6,Insl5,Stac,Smad9,Slc34a1,Fam19a1
FREAC2_01	0.005	0.0203	Pcsk1,Neurod6,Smad9,Slc30a8,Tmprss15,Fam19a1
CHX10_01	0.0116	0.0325	Krt73,Itgbl1,Stac,Fam71f1,Fam19a1
MYOD_Q6	0.0207	0.047	Ppp1r17,Gfi1,Nhlh2,Neurod6,Fam71f1

### Hydrophobicity profiling of protein

**Figure 8:**
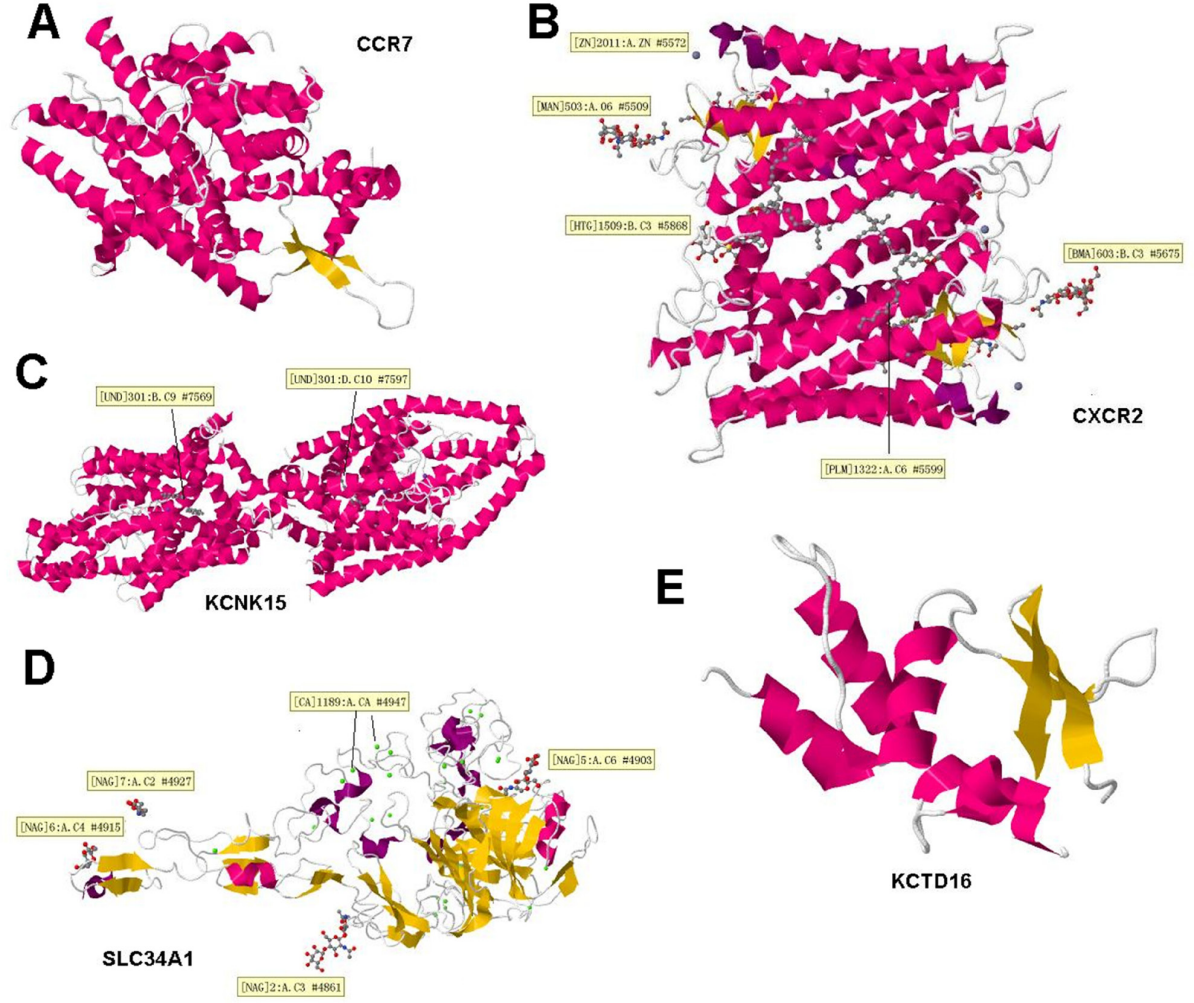
3D structure of four selected proteins Words in the yellow boxes were the information about the active binding sites. In the 3D structures, red ones mean α-helix, the orange ones mean β-fold. (**A**–**E**) represents CCR7, CXCR2, KCNK15, SLC34A1, KCTD16.

Gene functional part is protein, and the localization of proteins determines its functional fate. Hence we carried out the hydrophobicity profiling and their localization profile. Therefore, to figure out the mechanistic grounds for the interacting genes we firstly performed hydrophobicity profiling of proteins. For this purpose we used ExPASy (http://web.expasy.org/protparam/) to calculate the Grand average of hydropathicity (GRAVY) for CCR7, CXCR2, KCNK15, SLC34A1, KCTD16, results were listed in Table 5. Furthermore, we find Primary, secondary and tertiary structure, did homology modeling, figure out important protein localization predictions and finally performed cytoplasmic vs nuclear protein-protein docking and displayed in the 3D structure of selected proteins (CCR7, CXCR2, KCNK15, SLC34A1, KCTD16), and found the ligands (if they have) which can dock and bind to the protein active sites as shown in Figure 8.

**Table 5:** GRAVY list of CCR7, CXCR2, KCNK15, SLC34A1, KCTD16

Gene	GRAVY
CCR7	0.613
CXCR2	0.647
KCNK15	0.027
SLC34A1	0.397
KCTD16	-0.677

## DISCUSSION

AMH suppress the growth of follicle development by inhibiting the development of primordial follicle to primary follicles transition [16, 17]. And it was also found that it can inhibit the primordial follicle transition stimulated by different growth factors in rats [14].

A computational biology research was used to elucidate the understanding of genes expression that could play important role in the transition of primordial to primary follicles in ovaries. In the present network analysis, we aimed to investigate the changes in transcriptome profile of preantral and small antral mouse follicles after culturing with AMH and thereby identify candidate genes and pathways to be involved. As AMH show inhibitory function in the recruitment of primordial follicular development, the female AMH treated model can give the useful information about the recruitment of follicles and the linkages between follicular dynamics and reproductive capabilities of ovaries [19]. In the current research mice ovaries were cultured with two time points (12 h and 24 h) and at two concentrations of AMH (50 ng/ml and 200 ng/ml) and the downstream gene were checked. In this duration there was no morphological change in the ovarian tissues in treated and –untreated groups indicating that the changes in the downstream transcriptome were due to the change in mRNAs which were affected by different time and concentration of AMH in cultured ovaries. Four sets of DEGs were identified from four groups, which were divided according to AMH treated time points and AMH concentrations. We showed specific gene expression patterns in each group by clustering genes and samples, then defined two gene sets by comparing among groups that are; DEGs connected with time points and DEGs connected with concentration of AMH. The further analysis were based on these two gene sets.

Gene set 1 which contained DEGs connected with time points, most of them were significantly related to cell adhesion, 23 AMH treated time points related DEGs directly participated in cell adhesion, such as AZGP1, PODXL2, DSG1A, PCDHB18, CDH18, etc. Cell adhesion is the process by which cells interacts and attach to a surface, substrate or another cell, mediated by interactions between molecules of the cell surface. Cell adhesion occurs from the action of transmembrane glycoproteins [20, 21]. As anti-Müllerian hormone (AMH) is a glycoprotein hormone structurally related to inhibin and activin belongs to the transforming growth factor beta superfamily. The primordial to primary follicle transition requires extensive changes in cell shape and size as the flattened pre-granulosa cells proliferate and become cuboidal, and the oocyte starts to enlarge. It is expected that cell adhesion molecules are involved in this process, and that the inhibitory actions of AMH would results in changes in cell adhesion gene activity compared to untreated controls. In addition, it is possible that AMH may be gradually transported into cells through targeting the DEGs which were related to cell adhesion. Among four enriched pathways, the cytokine-cytokine receptor interaction (mmu04060) and Chemokine signaling pathway (mmu04062) take our attention, which shared 2 genes: CCR7, CXCR2. AMH regulates these receptors, upon which, after a series of putative conformational changes and phosphorylation steps, gene expression is regulated in the cell [22, 23]. Chemokines are small peptides that act through limited number of receptors and play an important role to maintain the normal physiology of different organs [9]. CCR7, CXCR2 belong to chemokine receptors, and AMH regulates these receptors and change the downstream gene expressions and cell physiology. Cytokine-cytokine receptor and neuroactive ligand-receptor pathways were also reported when rat ovaries were treated with 50 ng/ml of AMH [24] indicating that different genes are using the same pathways to affect the follicle development in different species.

Set 2 genes contained DEGs connected with concentrations of AMH, DEGs: KCNK15, SLC34A1, KCTD16, SLC30A8, 1300017J02RIK showed the same trend of taking part in three BP terms at the same time: metal ion transport, ion transport and cation transport. These genes involved in process of ion transportation have a predominant role in development of an organism and are believed to be putative target of AMH. The present study provided a new group of genes to be investigated in the development of follicular growth by interacting the ion transport pathways of an organism which could change the cell shape or physiology. Moreover, some important genes in TF-miRNA networks were up regulated or down regulated i.e., SMAD9 node (Figure 6B) which belong to TGF-β signaling, and are regulated in response to AMH treatment which validate our network results.

Furthermore, the potential of inhibition or stimulation of primordial follicle in a therapeutic treatment shows several clinical applications. An interval in the primordial follicle development and preservation of the primordial follicles could play a critical role in the prolongation of the reproductive life of a female. Moreover, the remedial inhibitory potential on the primordial follicle development could be used as a treatment for the premature ovarian failure, when the ovaries lost the primordial follicle pool resulting in early female infertility. In this process a complex cellular interaction is required for the balanced transition of primordial to primary follicle. The molecular control of primordial pool and the transition of primordial to primary follicle contribute the information about the regulation of ovarian function and may lead to treatments of ovarian diseases [25]. The stimulation of primordial follicle development could also cause loss in the primordial follicular pool and induce sterility. A number of pathways and gene interaction may contribute towards induced sterility. The computational analysis and intra-cellular and extra-cellular protein docking was performed in the present study to provide mechanistic grounds for such events and provide therapeutic targets for further research to prevent induced sterility.

## MATERIALS AND METHODS

### Data set and description

We downloaded the gene expression profiles from GEO (Gene Expression Omnibus, http://www.ncbi.nlm.nih.gov/geo/) with the accession number GSE56737, which contained 18 samples in total (platform: GPL1261 [Mouse430_2] Affymetrix Mouse Genome 430 2.0 Array). Microarray was the first high throughput technology that was developed to measure more than twenty thousand genes at the same time in a given sample [26].

For the initial studies that produced these datasets, preantral to small antral follicles were collected from dissected ovaries of seven to eight weeks old female C57BL/6Tac mice. In each experiment, follicles were pooled to obtain single biological sample and for each set three physiological replicates were used. Twelve hour as well as 24 hour experiments were performed with two different AMH concentrations. Human recombinant anti-müllerian hormone (rh-AMH) (R & D Systems, USA) at a concentration range of 0, 50 and 200 ng/ml was used in this research. In addition, the cultured medium was also supplemented with 80 IU/L Follicular stimulating hormone (FSH) and 10 IU/L of Luteinizing hormone (LH) (Sigma, USA). The same procedure was used in all experiment.

The total RNA was extracted by RNAasy micro kit and the concentration and quality of RNA of each sample was checked by Bioanalyzer 2100. Later on, 3.7 ug of fragmented cRNA was loaded on the Affy MG 430 2.0 probe array cartridge and was hybridized according to standard protocol, and arrays were scanned at 560 nm by using confocal laser scanning microscope (Affy Scanner 3000 7G).

### Data processing and DEGs screening

Raw CEL files and the probe annotation files were downloaded, and the gene expression data of all samples were preprocessed via background correction, quantile normalization and probe summarization using the Robust Multi-array Average (RMA) algorithm in Affy software package of Bioconductor (available at http://www.bioconductor.org/packages/release/bioc/html/affy.html) [27]. Here, we divided samples into different groups and defined them as follows: group a. AMH 200 ng/ml 12 h; group b: AMH 50 ng/ml 12 h; group c. AMH 200 ng/ml 24 h; group d. AMH 50 ng/ml 24 h. The Linear Models for Microarray Data (LIMMA) package [28] of Bioconductor was used (http://www.bioconductor.org/packages/release/bioc/html/limma.html) to identify differentially expressed genes (DEGs) in each group compared to their respective 0 ng/ml controls (a and b compared to 0 ng/ml 12 h; c and d compared to 0 ng/ml 24 h). We compared group a DEGs vs b, group c DEG's vs d, group a DEGs vs c, and group b DEGs vs d. In each group, only the genes meeting FDR < 0.05 and|log_2_FC (fold change)|>1were chosen as DEGs. (graphical display for different group and gene set definition was shown in Figure 4).

### Hierarchical clustering and comparison analysis of selected DEGs in different groups

The expression of selected DEGs between different versus groups were used to generate hierarchical clustering images by pheatmap package in R [29], respectively (https://cran.r-project.org/web/packages/pheatmap/). Then, we compared selected DEGs in group a, b, c and d groups, and got overlapped genes in each group by drawing VENN diagram, using VennDiagram package (https://cran.r-project.org/web/packages/VennDiagram/) in R [30]. Here, we defined two gene sets which would be further analyzed: Gene set 1: contained overlapped DEGs between a and b plus overlapped DEGs between c and d, defined as DEGs connected with time points. Gene set 2: contained overlapped DEGs between a and c plus overlapped DEGs between b and d, defined as DEGs connected with concentrations of AMH (graphical display for different group and gene set definition was shown in Figure 4).

### Enrichment analysis for DEGs in gene set 1 and 2

To explore the functions of DEGs in gene set 1 and 2, the DAVID (Database for Annotation, Visualization and Integrated Discovery, https://david.ncifcrf.gov/) database [31, 32] was used to perform GO (Go Ontology) [33] and KEGG (Kyoto Encyclopedia of Genes and Genomes) [34] pathway enrichment analyses for co-regulated DEGs. The *p*-value < 0.05 and gene count ≥2 were set as the cut-off criteria. Furthermore, the category of enriched GO, KEGG terms and the gene number were displayed as a histogram which was constructed by ggplot2 package in R [35] (https://cran.r-project.org/web/packages/ggplot2/).

### Co-expression network analysis for DEGs in gene set 1 and 2

We used cor function in R language to calculate co-expression Pearson Correlation Coefficient (PCC) [36] among DEGs in gene set 1 and set 2, respectively. We kept gene pairs whose PCC score were over than 0.8 as co-expression gene pairs, and displayed co-expression network by utilizing Cytoscape3.2.0 [37] (http://www.cytoscape.org/).

### miRNA-DEGs-TF target regulatory network analysis

**Figure 9:**
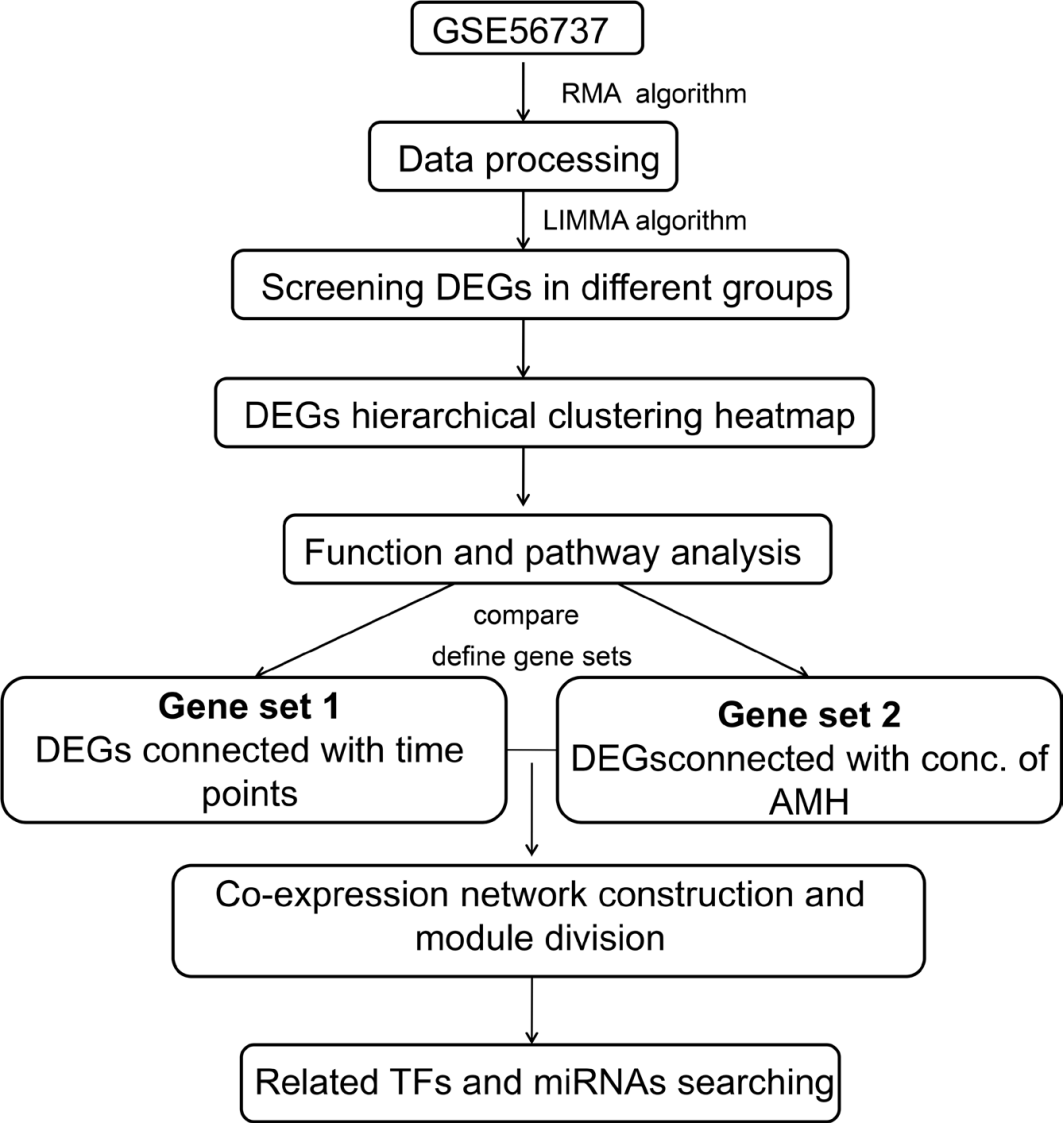
Schematic illustration of the analysis strategy

WebGestalt (WEB-based Gene SeT AnaLysis Toolket) (http://www.webgestalt.org/option.php) [38] database, which is a functional enrichment analysis web tool, to predict TFs and miRNAs which regulated the DEGs in co-expression networks of gene set 1 and 2. *P*-value < 0.05 was set as the significance cut-off criteria. We then integrated results of TFs and miRNAs, constructed miRNA-DEGs-TF regulatory network. The regulatory network consisting of DEGs, miRNAs and TFs were then visualized by Cytoscape3.2.0 [37] (http://www.cytoscape.org/). A schematic diagram of the overall procedure for analysis is presented in Figure 9.

## CONCLUSIONS

In summary, we figured out DEGs influenced by AMH from two different aspects: treatment time and AMH concentration. They may help us in the understanding about the AMH action in the regulation of gene expressions in ovarian follicles. It was assumed that different treatment time may affect cell adhesion related gene expressions, and so affect follicular changes in cell shape or AMH transmembrane transport with receptors. Different concentrations of AMH may change ion transport capacities of genes. This analysis research demonstrates some potential mechanism which may inhibit the transition of primordial follicle to primary follicles. The DEGs results show previously unidentified pathways and signaling factors that could regulate the transition of primordial to primary follicles. Additional investigations are required to find the mechanism which these factors follow for the follicular regulation. The new finding may give some new insights that could lead to prolong the reproductive life of humans and can cure some infertility problems.

## SUPPLEMENTARY MATERIALS TABLES












