# Supplementary material for: Transcriptome profiling of anti-müllerian hormone treated preantral/small antral mouse ovary follicles
Source: Oncotarget. 2018 Jul 13;9(54):30253–67. doi: 10.18632/oncotarget.25572 (PMC6084392; doi:10.18632/oncotarget.25572)
Supplement: Supplementary file 1 [file oncotarget-09-30253-s001.pdf]

Transcriptome profiling of anti-müllerian hormone treated preantral/small antral mouse ovary follicles

SUPPLEMENTARY MATERIALS

Supplementary Table 1: Data set GSE56737 at concentrations 50 and 200 ng at 12 and 24 hours interval. See Supplementary_Table_1

Supplementary Table 2: List of differences at 12 and 24 hours' time intervals at 50 and 200 ng concentrations intervals. See Supplementary_Table_2

Supplementary Table 3: List of Gene set 1 and Gene set 2 logFCa&b, logFCc&d, logFCa&c, logFCb&d. See Supplementary_Table_3

Supplementary Table 4: List of Gene set 1 and Gene set 2 cor 1-0.8 values. See Supplementary_Table_4

Supplementary Table 5: List of Gene set 1 and Gene set 2-Transcription Factors (TF) network. See Supplementary_Table_5
